# Reduced Graphene Oxide Sheets as Inhibitors of the Photochemical Reactions of α-Lipoic Acid in the Presence of Ag and Au Nanoparticles

**DOI:** 10.3390/nano10112238

**Published:** 2020-11-11

**Authors:** N’ghaya Toulbe, Malvina S. Stroe, Monica Daescu, Radu Cercel, Alin Mogos, Daniela Dragoman, Marcela Socol, Ionel Mercioniu, Mihaela Baibarac

**Affiliations:** 1 National Institute of Materials Physics, Laboratory of Optical Processes in Nanostructure Materials, Atomistilor str. 405 A, 77125 Bucharest, Romania; toulbe.nghaya@infim.ro (N.T.); malvina@infim.ro (M.S.S.); monica.daescu@infim.ro (M.D.); radu.cercel@infim.ro (R.C.); marcela.socol@infim.ro (M.S.); 2 Interdisciplinary School of Doctoral Studies, University of Bucharest, Șoseaua Panduri 90, 050663 Bucharest, Romania; 3 S.C. Agilrom Scientific S.R.L., 77190 Bucharest, Romania; alin.mogos@agilrom.ro; 4 Faculty of Physics, University of Bucharest, Șoseaua Panduri 90, București 050663, Bucharest, P.O. Box MG-11, 077125 Bucharest-Magurele, Romania; danieladragoman@yahoo.com or; 5 National Institute of Materials Physics, Atomic Structures and Defects in Advanced Materials Laboratory, Atomistilor str. 405 A, 77125 Bucharest, Romania; imercioniu@infim.ro

**Keywords:** α-lipoic acid, UV-VIS spectroscopy, SERS spectroscopy

## Abstract

The influence of Ag and Au nanoparticles and reduced graphene oxide (RGO) sheets on the photodegradation of α-lipoic acid (ALA) was determined by UV-VIS spectroscopy. The ALA photodegradation was explained by considering the affinity of thiol groups for the metallic nanoparticles synthesized in the presence of trisodium citrate. The presence of excipients did not induce further changes when ALA interacts with Ag and Au nanoparticles with sizes of 5 and 10 nm by exposure to UV light. Compared to the Raman spectrum of ALA powder, changes in Raman lines’ position and relative intensities when ALA has interacted with films obtained from Au nanoparticles with sizes between 5 and 50 nm were significant. These changes were explained by considering the chemical mechanism of surface-enhanced Raman scattering (SERS) spectroscopy. The photodegradation of ALA that had interacted with metallic nanoparticles was inhibited in the presence of RGO sheets.

## 1. Introduction

α-Lipoic acid (ALA) is present in various organisms of the human body, e.g., the kidney, heart, liver, and foods such as broccoli, spinach, and yeast extract [1]. The molecular structure of ALA contains both a carboxylic group and a dithiolane ring, and, depending on the chirality of the substituted carbon atom of the dithiolane ring, two enantiomers are known at present for this compound, i.e., RLA and SLA. ALA is used as a drug in diabetic neuropathy [2], as a dietary supplement to slow down the aging process of the body [3], and in obesity treatment [4]. The most important effect of ALA is its antioxidative capacity [5]. An improvement in ALA’s antioxidative effects has been reported in the presence of the Coenzyme Q10 (CQ10) [5]. Pharmaceutical compounds containing ALA are marketed under the name Alpha Lipoic Sustain, Tiolin, Alanerv, Alasod 600, and Cerebinox, the last of which also contains CQ10.

Until now, the main methods used in the characterization of ALA were (i) FTIR spectroscopy [6]; (ii) Raman scattering [6], (iii) UV-VIS spectroscopy [7], and (iv) cyclic voltammetry or square-wave voltammetry [8]. The detection of ALA was achieved using (i) electrochemistry methods [9], (ii) liquid chromatography [10], (iii) mass spectrometry [11], and optical methods based on surface plasmon resonance [12]. A recent application of metallic nanoparticles that have interacted with ALA was used as a sensorial platform for nerve agents [13].

Considering the applications of ALA in the health domain, in this work, special attention is paid to the photodegradation process of ALA in the absence and the presence of metallic nanoparticles. The dependence of the photodegradation process of ALA in the presence of Ag and Au nanoparticles with various sizes is studied, too. The photodegradation process is also analyzed in pharmaceutical compounds such as Alpha Lipoic Sustain and Cerebinox. Using UV-VIS spectroscopy, we demonstrate that inhibition of ALA’s photodegradation that has interacted with Ag and Au nanoparticles can be induced in the presence of reduced graphene oxide (RGO) sheets. The differences between the Raman spectrum of ALA in the powder state and the surface-enhanced Raman scattering (SERS) spectrum of ALA as a thin layer deposited on rough films, obtained from the colloidal solutions of Au nanoparticles with sizes of 5, 10, and 50 nm, are also reported. A sustained effort was made to develop SERS supports by various methods such as (i) the oxidation/electrochemical reduction of metal electrodes, (ii) evaporation by the vacuum deposition of shaved metal films, (iii) lithography, and (iv) synthesis of colloidal metallic structures [14,15]. The two mechanisms that underlie the SERS effect, i.e., electromagnetic and chemical mechanisms, allow us to understand these differences. As is well known, the electromagnetic enhancement process of the Raman spectra is induced by the excitation of localized or delocalized surface plasmons generated at the dielectric/metal interface, while the chemical process involves the generation of new chemical bonds at the adsorbed molecule/metal interface [14]. Generally, SERS supports are characterized by rough structures with sizes in the range of 10–100 nm [15]. In order to highlight this property for the films obtained from the colloidal solution of Au nanoparticles, atomic force microscopy (AFM) studies are reported. The changes induced on the roughness parameters by the adsorption of RGO sheets onto Au films are also shown.

## 2. Materials and Methods

ALA (C_8_H_14_O_2_S_2_), sodium borohydride (NaBH_4_), trisodium citrate (Na_3_C_6_H_5_O_7_), silver nitrate (AgNO_3_), sodium hydroxide (NaOH), sodium citrate (C₆H₅Na₃O₇), tannic acid (C₇₆H₅₂O₄₆), tetrachloroauric acid (HAuCl_4_), potassium carbonate (K_2_CO_3_), and dimethylformamide (DMF, with 0.1% water) were purchased from the Aldrich-Sigma company (St. Louis, MO, USA). Pharmaceutical compounds, Alpha Lipoic Sustain and Cerebinox, were purchased from a local pharmacy (Jarrow Formulas, Inc., Los Angeles, CA, USA and Polisano Pharmaceuticals, Sibiu, Romania). The Alpha Lipoic Sustain pharmaceutical product contains, as active compounds, 300 mg of ALA and 333 mg of Biotin and, as additional ingredients, polyacrylic acid, Ca_3_(PO_4_)_2_, C₁₈H₃₆O₂, C₃₆H₇₀MgO₄, SiO_2_, cellulose, beetroot powder (for color), natural vanilla flavor, and a food-grade coating. The Cerebinox pharmaceutical product contains 300 mg of ALA, 10 mg of CQ10, 100 mg of wheat germ extract, caking, and SiO_2_.

The Ag nanoparticles with an average size of cca. 5 and 10 nm were prepared according to Reference [16]. Briefly, to obtain Ag nanoparticles with a size of 5 nm, mixing by vigorous stirring in the dark of a 48 mL aqueous solution containing 2 mM NaBH_4_ and 4.28 mM Na_3_C_6_H_5_O_7_ at 60 °C, for 30 min, was carried out. After that, 2 mL of 1 mM AgNO_3_ was added to the above solution. Further, the reaction mixture was preserved at 90 °C for another 20 min, the pH of the reaction mixture was adjusted to 10.5 using a 0.1 M NaOH solution. The yellow suspension of Ag nanoparticles was cooled to 25 °C and then centrifuged at 12,000 rpm for 15 min in order to remove all unreacted compounds. This step was followed by washing and redispersion in distilled water. A similar protocol was used to obtain Ag nanoparticles with a size of 10 nm, the only difference being the addition of 2 mL of 1.17 mM AgNO_3_ to the 48 mL solution containing 2 mM NaBH_4_ and 4.28 mM Na_3_C_6_H_5_O_7_. According to Reference [16], the particle concentration in the colloidal solutions of Ag with particle sizes of 5 and 10 nm was equal to 1.03 × 10^15^ and 1.53 × 10^14^ particles/mL, respectively.

Au nanoparticles with average sizes of cca. 5 and 10 nm were synthesized using the protocol described in Reference [17]. The synthesis protocol involves (i) the preparation of a solution containing 150 mL of 2.2 mM Na_3_C_6_H_5_O_7_ and 0.01 mL of 2.5 mM C_76_H_50_O_46_, which was heated at 70 °C, and (ii) the addition of 1 mL of 25 mM HAuCl_4_ to the above solution, when one observes that, after cca. 10 s, the uncolored solution becomes orange-red. The addition of 1 mL of 150 mM K_2_CO_3_ to the above reaction mixture induced a change in pH from 10.4 to 8.3. This reaction mixture was preserved at 100 °C, for a period of 5 min, until a suspension of Au nanoparticles with a size of around 5 nm resulted [17]. In order to obtain Au nanoparticles with a size of cca. 10 nm, the only difference in the above protocol needed is the use of 0.01 mL of 2.5 mM tannic acid [17]. In the case of the colloidal solutions of Au with particle sizes of 5 and 10 nm, the particle concentration was equal to 4.4 × 10^13^ and 1.2 × 10^13^ particles/mL [17]. In References [16] and [17], TEM studies demonstrated that these protocols for the preparation of the metallic nanoparticles reported led to (i) Ag nanoparticles with size ranges of 5 ± 0.7 nm and 10 ± 2.0 nm [16] and (ii) Au nanoparticles with size ranges of 5.1 ± 0.5 nm and 10.5 ± 0.9 nm [17].

The Au nanoparticles with a size of cca. 50 nm were synthesized by the addition of 1 mL of 1wt.% HAuCl_4_ to 47 mL of water, and the mixture was heated to 100 °C when 1.5 mL of Na_3_C_6_H_5_O_7_ were injected [18,19]. The reaction mixture was preserved at 100 °C for 45 min, until its color became red, and was then cooled at 25 °C [18,19]. The colloidal solution of Au, with a particle size of 50 nm, had a particle concentration equal to 1.05 × 10^11^ particles/mL [19]. For the SERS studies, the rough Au films were obtained by the evaporation of a 0.12 mL colloidal solution of Au with a particle size of 50 nm. The roughness parameters of these Au films before and after the deposition of RGO sheets were evaluated by atomic force microscopy (AFM).

The AFM images were collected (in phase feedback) with a Nanonics Multiview 4000 microscope (Nanonics Imaging Ltd., Jerusalem, Israel) working in tapping mode. We used a cantilever with a 20 nm diameter, a 1730 factor of merit, and a 34.7 kHz vibration frequency.

The photodegradation processes were monitored by UV-VIS spectroscopy. In this order, solutions of ALA in (i) DMF, (ii) a stabilized suspension in a citrate buffer of Au nanoparticles with diameters equal to 5 and 10 nm, respectively, and (iii) the dispersions of the citrate functionalized Ag nanospheres with sizes equal to 5 and 10 nm, respectively, in aqueous sodium citrate, were used. The solutions of ALA or pharmaceutical products used in the UV-VIS spectroscopy studies had a 1 mg/mL concentration. All spectra were recorded with a Perkin Elmer UV-VIS-NIR spectrophotometer, Lambda 950 model (PerkinElmer, Inc., Waltham, MA, USA), in the 200–600 nm spectral range, the scan rate being 96 nm/min. The UV-VIS spectra of each sample were recorded at intervals of 5 min of UV irradiation for a period of 60 min. UV irradiation was performed using a Hg-vapors lamp with a power of 350 W.

The Raman spectra of ALA were recorded with an FT Raman spectrophotometer, model RFS100S (Bruker Optik GmbH, Ettlingen, Germany). The concentration of ALA solutions was 10^−2^ M for the Raman scattering studies. The films obtained from the colloidal solution of Au, with a particle size of 50 nm, were prepared on quartz substrates with an area of 1 cm^2^.

Samples for transmission electron microscopy (TEM) and high-resolution transmission electron microscopy (HRTEM) were prepared by suspending them in ethanol and transferring them to a copper grid coated with amorphous carbon support. TEM and HRTEM images were recorded with a JEOL JEM ARM 200 F electron microscope (JEOL (Europe) SAS) operated at 200 keV.

A solution consisting of 0.5 mg of RGO in 1 mL of DMF was prepared to highlight the influence of the RGO sheets on the roughness parameters of Au films.

## 3. Results and Discussion

### 3.1. The Photodegradation of ALA Highlighted by UV-VIS Spectroscopy

**Figure 1:**
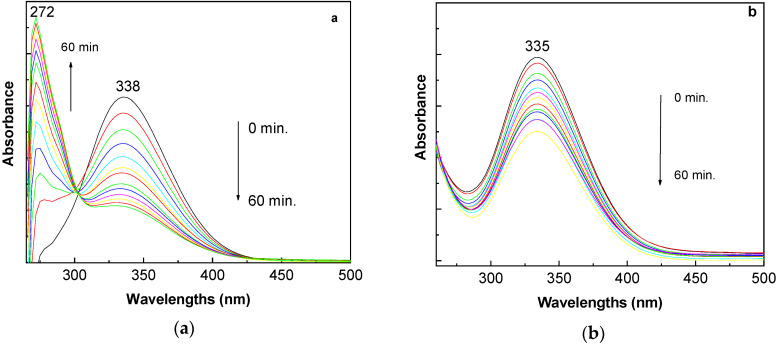
UV-VIS spectra of α-lipoic acid (ALA) in dimethyl formamide (DMF) (**a**) and in the mixture of DMF–H_2_O with the volume ratio equal to 1:1 (**b**). The concentration of ALA is 1 mg/mL. The two samples’ UV-VIS spectra were recorded at intervals of 5 min of exposure to UV light, for 60 min.

**Scheme 1:**
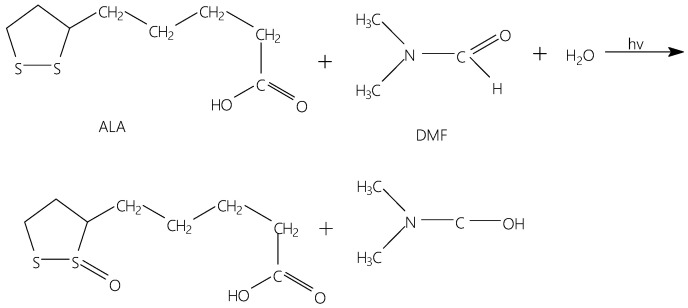
The photochemical reaction of ALA in the presence of DMF and H_2_O.

Figure 1a shows the UV-VIS spectrum of ALA in DMF in the initial state (black curve), characterized by a band with a maximum at 338 nm. The exposure of the sample at the UV light led to a decrease in the absorbance of this band, simultaneous with the appearance and the increase in the band’s absorbance at 272 nm, a modification that involved the appearance of an isosbestic point at 300 nm. This suggests that new chemical compounds are generated under UV light in a solution of ALA in DMF containing 0.1 wt.% water, according to Scheme 1. In the case of a semi-aqueous ALA solution, the exposure to UV light induced only a decrease in the band’s absorbance at 335 nm (Figure 1b).

**Figure 2:**
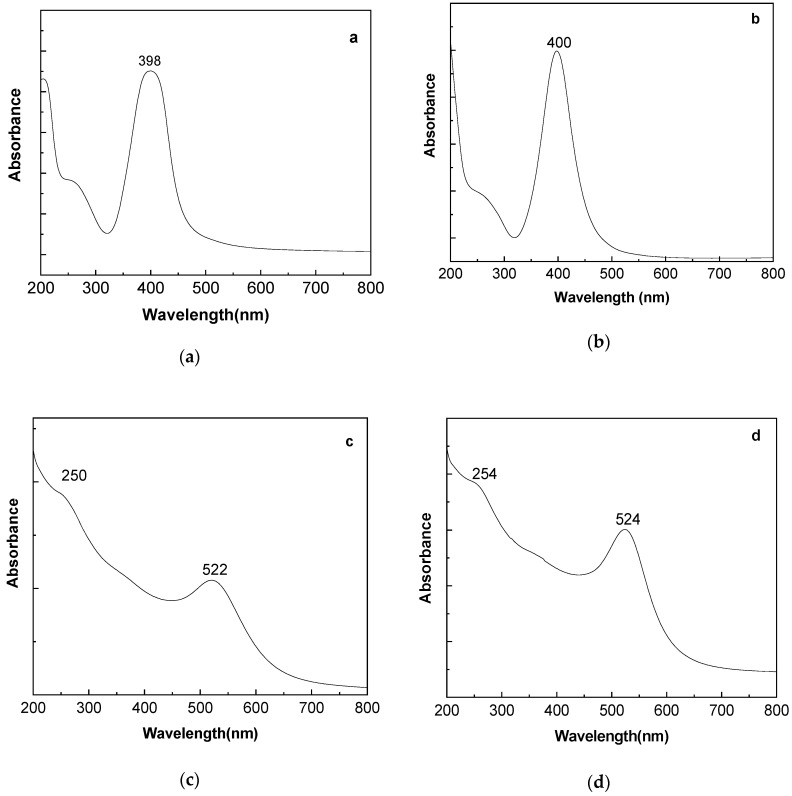
UV-VIS spectra of nanoparticles: (**a**) Ag with a particle size of 5 nm, (**b**) Ag with a particle size of 10 nm, (**c**) Au with a particle size of 5 nm, and (**d**) Au with a particle size of 10 nm.

**Figure 3:**
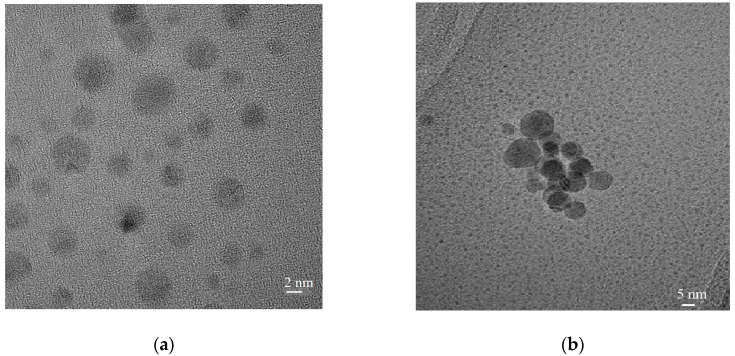

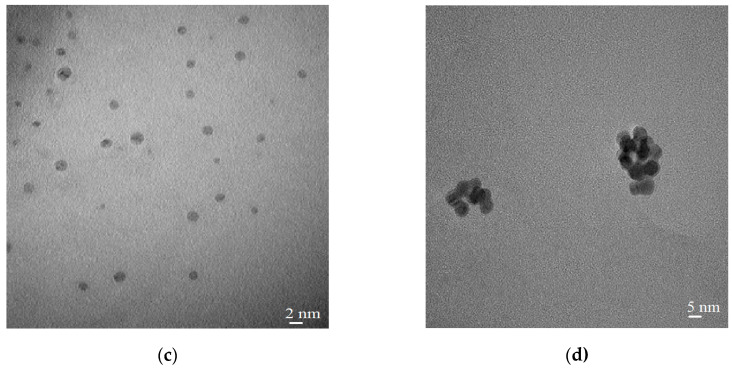
HRTEM images of the nanoparticles: (**a**) Ag with a particle size of 5 nm, (**b**) Ag with a particle size of 10 nm, (**c**) Au with a particle size of 5 nm, and (**d**) Au with a particle size of 10 nm.

The UV-VIS spectra of Ag and Au nanoparticles must be shown to assess the influence of metallic nanoparticles on ALA. In this context, Figure 2 highlights that the plasmonic bands of the Ag nanoparticles with sizes of 5 nm and 10 nm peaked at 398 nm (Figure 2a) and 400 nm (Figure 2b), while those of the Au nanoparticles with sizes of 5 nm and 10 nm were situated at 522 nm (Figure 2c) and 524 nm (Figure 2d). Figure 3 shows the HRTEM images of the metallic nanoparticles. According to Figure 3, regardless of the Ag and Au nanoparticles’ size, they show a spherical shape. The UV-VIS spectra show relatively broad bands, which indicates a broad size distribution. This fact is confirmed by the TEM images shown in Figure 3.

**Figure 4:**
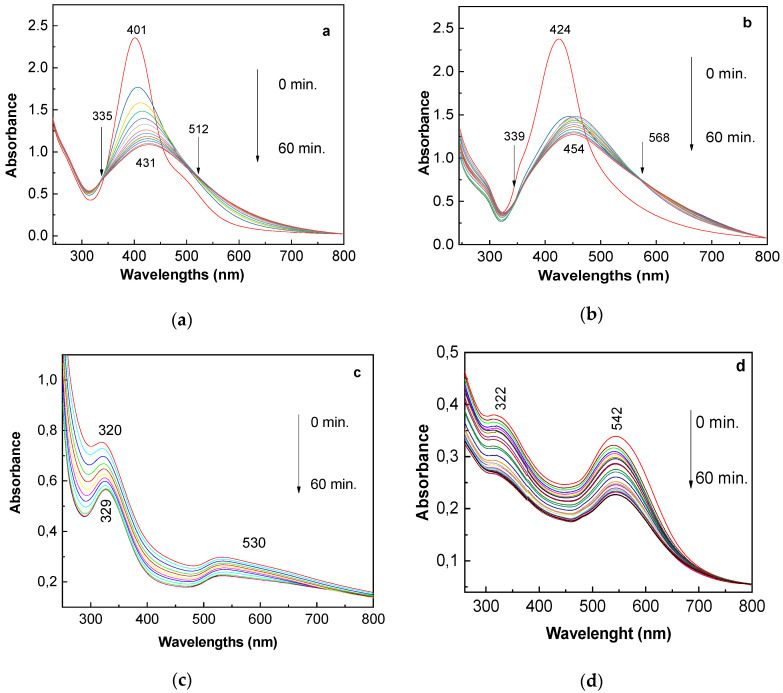
UV-VIS spectra of ALA in the presence of Ag with a particle size of 5 nm (**a**), Ag with a particle size of 10 nm (**b**), Au with a particle size of 5 nm (**c**), and Au with a particle size of 10 nm (**d**). The concentration of ALA is 1 mg/mL. The four samples’ UV-VIS spectra were recorded at intervals of 5 min of exposure to UV light, for 60 min.

Figure 4 shows the UV-VIS spectra of ALA that has interacted with Au and Ag nanoparticles, both with sizes of 5 and 10 nm, the ALA concentration in the aqueous solution of metallic nanoparticles being equal to 1 mg/mL. According to Figure 4 (red curves), in the initial state, i.e., before exposure to UV light, the UV-VIS spectra were characterized by absorption bands with maxima at 322 nm and 542 nm in the case of ALA that has interacted with Au nanoparticles with a size of 10 nm (Figure 4d). These bands were situated at 320 nm and 530 nm in the case of ALA that has interacted with Au nanoparticles with a size of 5 nm (Figure 4c). In the case of ALA that has interacted with Ag nanoparticles with sizes of 10 nm and 5 nm, the UV-VIS spectra show absorption bands with maxima at 424 nm (Figure 4b) and 401 nm (Figure 4a), respectively. The exposure of these samples to UV light for 60 min led to a gradual decrease in the absorbance of the band localized in the spectral range 300–700 nm (Figure 4). Supplementarily, in the case of ALA that has interacted with Ag nanoparticles with sizes of 10 and 5 nm, one observes the following: (i) an up-shift of the absorption band at 454 nm (Figure 4b) and 431 nm (Figure 4a), respectively; (ii) the appearance of isosbestic points at 339 nm and 568 nm in the case of ALA that has interacted with Ag nanoparticles with a size of 10 nm (Figure 4b); and (iii) the appearance of isosbestic points at 335 nm and 512 nm in the case of ALA that has interacted with Ag nanoparticles with a size of 5 nm (Figure 4a). Regardless of the type of nanoparticles, i.e., Ag or Au, these results suggest development of photochemical reactions between ALA and metallic nanostructures. Considering the decreased absorbance of UV-VIS spectra, the photochemical processes between ALA and metallic nanoparticles were more intense in Ag nanoparticles, compared to Au nanoparticles. The more pronounced shift of the absorption band in the case of Ag nanoparticles that have interacted with ALA (Figure 4a,b) is believed to have its origin in the process of the chemical adsorption of ALA onto the surface of metal particles. Such a process would involve obtaining a more stable electronic state, i.e., the transformation of Ag into Ag^+^ (from [Kr] 4d^10^5s^1^ to [Kr] 4d^10^) and of Au into Au^+^ (from [Xe]4f^14^5d^10^ 6s^1^ to [Xe]4f^14^5d^10^]), for which it is known that the ionization energy is equal to 7.57 eV and 9.22 eV, respectively [20]. This fact was considered for the higher affinity of thiol groups of ALA for Ag nanoparticles compared with that for Au nanoparticles. Ag nanoparticles’ transformation into Ag_2_S was reported in 2017 when the appearance of non-stoichiometry in Ag sublattices was envisaged [21,22]. The formation of the thiolate-gold clusters is explained by several models reported by (i) Walter et al., who took into account the magic numbers of free valence electrons [23], and (ii) Cheng et al., who considered the superatom network and the super valence bond mode [24].

**Figure 5:**
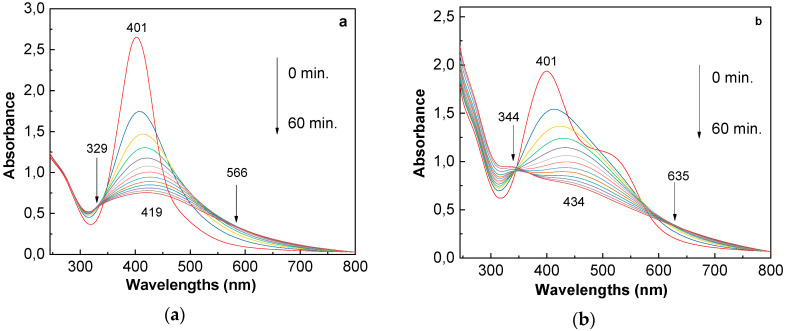

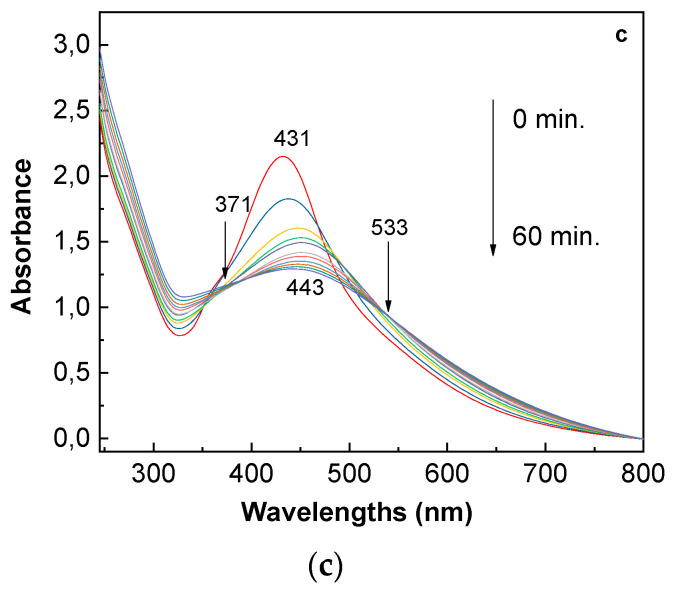
UV-VIS spectra of the ALA solution in the presence of excipients (commercial product marketed under the name Cerebinox) and Ag nanoparticles with a size of 5 nm, having an analyte concentration equal to 0.25 mg/mL (**a**) and 1 mg/mL (**b**), and their changes at exposure to UV light. Figure (**c**) shows the UV-VIS spectra of the Cerebinox solution with a concentration of 1 mg/mL in the presence of Ag nanoparticles with a size 10 nm and its change at exposure to UV light. The three samples’ UV-VIS spectra were recorded at intervals of 5 min of exposure to UV light, for 60 min.

**Figure 6:**
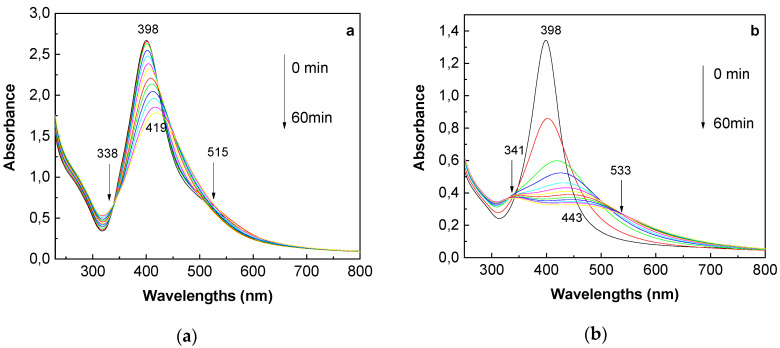
UV-VIS spectra of the ALA solution in the presence of excipients (commercial product marketed under the name of Alpha Lipoic Sustain) in the presence of Ag nanoparticles with sizes of 5 nm (**a**) and 10 nm (**b**), with a concentration of 1 mg/mL, and their changes at the exposure to UV light. The two samples’ UV-VIS spectra were recorded at intervals of 5 min of exposure to UV light for 60 min.

Figure 5 and Figure 6 show the influence of the additional active compounds to ALA and excipients. In this context, Figure 5 highlights the influence of the pharmaceutical compound concentration and the Ag nanoparticle size on the photochemical processes found in UV-VIS spectroscopy studies.

Figure 5b highlights that, in the case of the Cerebinox solution with a concentration of 1 mg/mL, prepared in the presence of Ag nanoparticles with a size of 5 nm, the UV-VIS spectrum shows an intense band with a maximum at 401 nm, whose absorbance decreases as the time of exposure to UV light increases, up to 60 min. This change was accompanied by the appearance of isosbestic points at 344 and 635 nm. The decrease in the concentration of Cerebinox in the solution of Ag nanoparticles with a size of 5 nm, at 0.25 mg/mL, induced an up-shift of the absorption band from 401 nm to 419 nm and the presence of isosbestic points situated at 329 and 566 nm (Figure 5a). The exposure of the Cerebinox solution with a concentration of 1 mg/mL to UV light for 60 min, prepared in the presence of Ag nanoparticles with a size of 10 nm, induced a shift in the band from 431 nm to 443 nm, which was simultaneous with the appearance of isosbestic points at 371 and 533 nm (Figure 5c). Similar behavior is evidenced in the pharmaceutical compound marketed under the name of Alpha Lipoic Sustain (Figure 6).

**Scheme 2:**
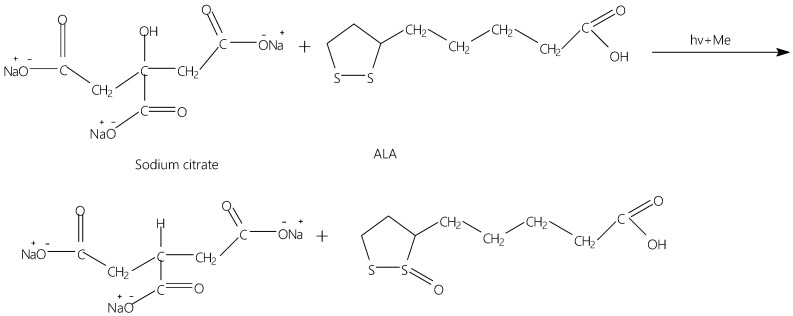
The photochemical reaction of ALA in the presence of metallic (Me) nanoparticles.

Thus, in the case of Alpha Lipoic Sustain solution, with a concentration of 1 mg/mL, (i) the exposure to UV light in the presence of Ag nanoparticles with a size of 5 nm induced a shift of the band from 398 nm to 419 nm, which was simultaneous with its decrease in absorbance and the appearance of isosbestic points at 338 nm and 515 nm. (ii) The exposure to UV light in Ag nanoparticles’ presence with a size of 10 nm led to a more significant decrease in the absorbance of the UV-VIS spectrum accompanied by a shift from 398 nm to 443 nm and the presence of isosbestic points at 341 nm and 533 nm. The results shown in Figure 5 and Figure 6 demonstrate that the presence of additional active compounds to ALA and the excipients do not influence the photochemical reaction of ALA with metallic (Me) nanoparticles. The origin of these spectral variations can be explained by taking into account the formation of new compounds in the presence of UV light, according to Scheme 2.

### 3.2. The RGO Sheets as Inhibitors of the ALA Photodegradation Highlighted by UV-VIS Spectroscopy

**Figure 7:**
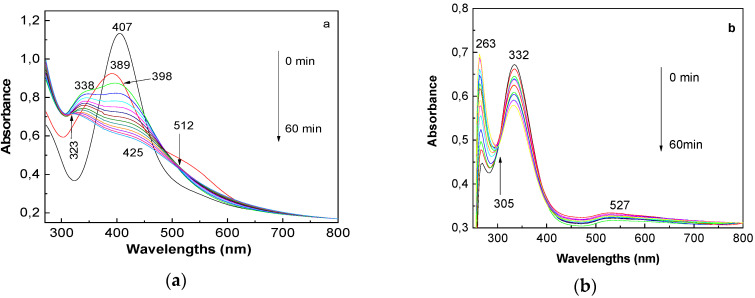
The influence of the UV light on the ALA solution resulting from the dissolution of 1 mg of ALA in the presence of (**a**) 0.5 mg RGO sheets in 1 mL of DMF and 1 mL of Ag nanoparticles with a size of 5 nm and (**b**) 0.5 mg RGO sheets in 1.5 mL of DMF and 0.5 mL of Ag nanoparticles with a size of 5 nm. The black curve in Figure **a** shows the UV-VIS spectrum of 0.5 mg RGO sheets in 1 mL of DMF and 1 mL of Ag nanoparticles with a size of 5 nm in the absence of ALA. The two samples’ UV-VIS spectra were recorded at intervals of 5 min of exposure to UV light, for 60 min.

**Figure 8:**
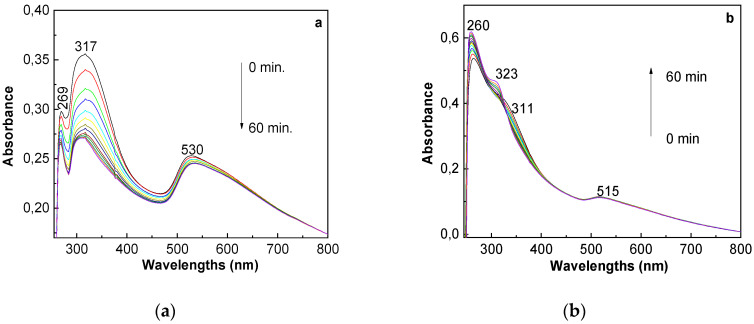
UV-VIS spectra of the ALA solution resulting from the dissolution of 0.5 mg in the presence of (**a**) 0.5 mg RGO in 0.5 mL of DMF and 1.5 mL of Au nanoparticles with a size of 5 nm and (**b**) 0.5 mg RGO in 1 mL of DMF and 1 mL of Au nanoparticles with a size of 5 nm, as well as their variations at exposure to UV light. The two samples’ UV-VIS spectra were recorded at intervals of 5 min of exposure to UV light, for 60 min.

Figure 7 and Figure 8 highlight the influence of RGO sheets on ALA’s photochemical reactions in the presence of metallic nanoparticles. According to Figure 7, the absorption band of the RGO sheets decorated with Ag nanoparticles with a size of 5 nm has a maximum at 407 nm. The UV-VIS spectrum of ALA in the presence of 0.5 mg RGO sheets in 1 mL of DMF and 1 mL of Ag nanoparticles with a size of 5 nm, before exposure to UV light, is characterized by a band with a maximum at 389 nm. According to Figure 7a, the exposure to UV light induced the following: (i) in the first 5 min, a shift of the band from 389 nm to 398 nm and the appearance of a new band observed as a shoulder at 338 nm; (ii) a gradual shift of the bands from 398 nm and 338 nm to 425 nm and 323 nm, respectively, when the time of UV irradiation varied from 5 min to 60 min; (iii) a decrease in the absorbance of the two bands at 398–425 nm and 338 nm, which involved a change in the ratio between their absorbance (A_398–425_/A_338_) from 1.05 to 0.83, when the exposure time at UV light increases from 5 to 60 min; and (iv) the appearance of an isosbestic point at 512 nm.

According to Figure 7b, the exposure of the ALA to UV light in the presence of 0.5 mg RGO sheets in 1.5 mL of DMF and 0.5 mL of Ag nanoparticles with a size of 5 nm induced a decrease in the absorbance of the band at 332 nm. This was simultaneous with the increase in the absorbance of the band at 263 nm and the appearance of an isosbestic point at 305 nm. A careful analysis of the band’s absorbance variation in the spectral range 300–500 nm highlights a decrease from (i) 0.92 to 0.6 in Figure 7a and (ii) 2.36 to 1.08 in Figure 3d. These results demonstrate the inhibitory role of RGO sheets on the photochemical reactions between ALA and Ag nanoparticles. Such behavior is also shown in the case of Au nanoparticles. Figure 8 is relevant in this context.

**Scheme 3:**
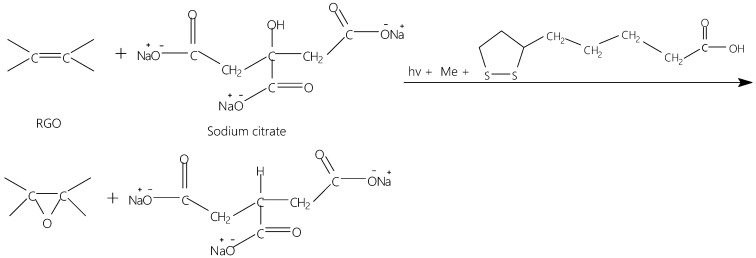
The interaction of RGO sheets with ALA and Me nanoparticles stabilized with sodium citrate.

The black curve in Figure 8a shows the UV-VIS spectrum of the ALA solution resulting from the dissolution of 0.5 mg of ALA in the presence of 0.5 mg RGO in 0.5 mL of DMF and 1.5 mL of Au nanoparticles with a size of 5 nm. Three bands with maxima at 269 nm, 317 nm, and 530 nm are shown in Figure 8a. According to Figure 8a, the exposure to UV light induced a decrease in the absorbance of bands at (i) 269 nm, from 0.3 to 0.26, (ii) 317 nm, from 0.36 to 0.27, and (iii) 530 nm, from 0.25 to 0.24. In the ALA solution resulting from the dissolution of 0.5 mg of ALA in the presence of 0.5 mg RGO in 1 mL of DMF and 1 mL of Au nanoparticles with a size of 5 nm, no change was observed in the absorbance of the band with a maximum at 515 nm (Figure 8b). The only change observed in Figure 8b regards the band’s shift from 311 nm to 323 nm, a variation accompanied by an increase in its absorbance from 0.41 to 0.47. An increase in the absorbance of the band at 260 nm from 0.54 to 0.62 was also reported. These values were significantly lower than those reported in Figure 5a. These results proved once again the inhibitory role of RGO sheets on the photochemical reaction between ALA and Au nanoparticles. An explanation for the inhibitory role of the RGO sheets on the photochemical reaction between ALA and Me nanoparticles is shown in Scheme 3.

### 3.3. Raman Scattering and SERS Spectroscopy Studies on ALA

**Figure 9:**
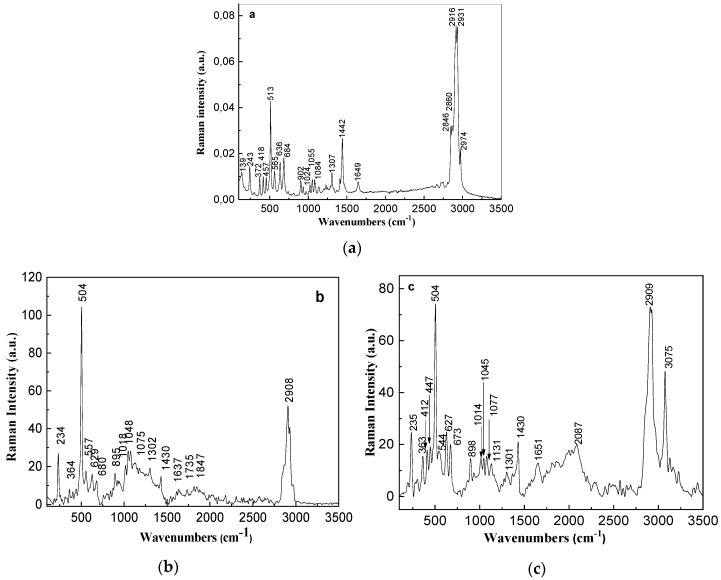

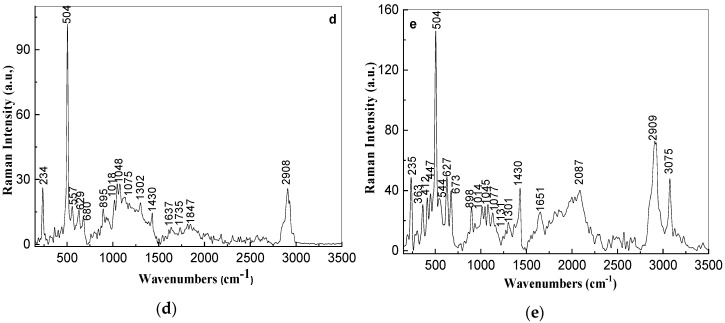
Raman spectra of ALA in a solid state (**a**) and as layers deposited onto metallic films prepared using colloidal solutions of Au nanoparticles with sizes of 5 nm (**b**) and 10 nm (**c**) and of Ag nanoparticles with sizes of 5 nm (**d**) and 10 nm (**e**). The ALA layers were obtained by using ALA solutions with a concentration of 10^−^^2^ M.

In order to prove the adsorption of ALA onto metallic nanoparticles functionalized with sodium citrate, Figure 9 shows the differences between the Raman spectra of ALA when this organic compound is in the form of a powder (Figure 9a) and a layer deposited onto Ag and Au films resulting from colloidal solutions of Ag and Au with particle sizes of 5 and 10 nm (Figure 9b–e). According to Figure 9a, the main Raman lines of ALA peaked at 243, 372–418–457, 513, 565, 636, 684, 902, 1024, 1055, 1084–1230, 1307, 1442, 1649, 2846–2860, and 2916–2931–2974 cm^−^^1^, assigned to the following vibrational modes: deformation ring, deformation CSSC, stretching S-S, stretching 1, 2-dithiolane ring, stretching C-S out-of-phase, stretching C-S in-phase, stretching C-C in 1, 2-dithiolane ring, stretching C-C, stretching C-C trans, in-plane deformation C-H in a heteroatomic ring, twisting of CH_2_ group, deformation scissoring of CH_2_ group, stretching C=O in amide, and anti-symmetrical stretching CH_2_ in alkyl chain/anti-symmetrical stretching CH_2_ in a 1,2-dithiolane ring. [2,25] The values of the ratio between Raman lines’ intensities peaked at 513, 1442, 1024, and 2916 cm^−^^1^, i.e., I_513_/I_2916_, I_1442_/I_2916_, and I_1024_/I_2916_ are equal to 0.57, 0.35, and 0.09, respectively. In the context of the discussion concerning the interaction of ALA with metallic nanoparticles, it is important to know that the ratio I_513_/I_684_ has a value of 2.36. In contrast to Figure 9a, the following vibrational changes are observed in Figure 9b,d, i.e., in the case of ALA layers deposited onto metallic films resulting from a colloidal solution of Ag nanoparticles with sizes of 5 and 10 nm: (i) a down-shift of the Raman lines from 513, 684, 1024, 1442, and 2916 cm^−^^1^ (Figure 9a) to 504, 684, 1018, 1430, and 2908 cm^−^^1^, respectively (Figure 9b,d); and (ii) a change in the value of the ratios I_513_/I_2916_, I_1442_/I_2916_, I_1024_/I_2916_, and I_513_/I_684_ from 2, 0.28, 0.4, and 8.35 (Figure 9b) to 3.94, 0.56, 0.79, and 8.2, respectively (Figure 9d). The changes in the intensities and the positions of Raman lines when the ALA layer is deposited onto metallic films resulting from the colloidal solutions of Au nanoparticles with sizes of 5 and 10 nm are noted in Figure 9c,e as follows: (i) the Raman lines from 513, 680, 1024, 1442, and 2916 cm^−^^1^ (Figure 9a) shifted to 504, 673, 1014, 1430, and 2909 cm^−^^1^, respectively (Figure 9c,e); (ii) in the 3000–3500 cm^−^^1^ spectral range, a new Raman line with a maximum at 3075 cm^−^^1^ appeared; (iii) values of the ratios I_504_/I_2909_, I_1430_/I_2909_, I_1014_/I_2909_, and I_504_/I_673_ changed from 1.01, 0.29, 0.2, and 3.7 (Figure 9c) to 2, 0.57, 0.4 and 3.67 (Figure 9e). 

The variations above indicate a chemical interaction between metallic nanoparticles functionalized with sodium citrate and ALA, as shown in Scheme 2. Regardless of the metallic nanoparticle type, i.e., Ag or Au, the increase in the Raman line’s intensity with a maximum at 504 cm^−^^1^ suggests preferential chemical adsorption of ALA onto metallic nanoparticles, as reported in the case of other thiols [25]. Evidence of the chemical adsorption of ALA onto the metallic nanoparticle surface is shown by the Raman line with a maximum at 234 cm^−^^1^, observed in Figure 9b–e, which was assigned to the stretching vibration mode of the metal-adsorbate bond [2]. The presence of these Raman lines in Figure 9b–e can be explained by considering the chemical mechanism of the SERS effect that involves a charge transfer at the metal/dielectric interface—in our case, the Ag or Au nanoparticles and ALA, when new bonds between the metal and adsorbate of the type Ag-S or Au-S are generated [25].

Using a protocol proposed by Laurent et al. [26] to assess the enhancement factor (EF) of the SERS process and the experimental results shown in Figure 9, the following EFs of ALA were calculated for the Raman line situated in the spectral range 500–520 cm^−^^1^: (i) 1.15 × 10^7^, in the case of Ag nanoparticles with sizes of 5 nm and 10 nm, (ii) 8.16 × 10^6^, in the case of Au nanoparticles with a size of 5 nm, and (iii) 1.6 × 10^7^, in the case of Au nanoparticles with a size of 10 nm.

**Figure 10:**
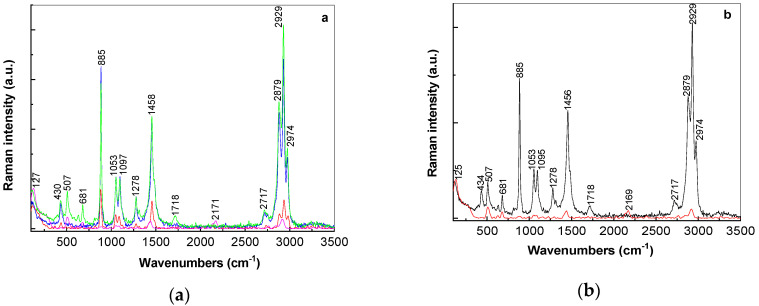
Raman spectra of the ALA layer deposited onto metallic films prepared using 1 mL (red curve), 2 mL (blue curve), 4 mL (magenta curve), and 6 mL (green curve) colloidal solutions of Au nanoparticles with a size of 50 nm (**a**). The dependence of the intensity of Raman spectra on the ALA layers deposited onto metallic films prepared from 6 mL colloidal solutions of Au nanoparticles with sizes of 50 nm (black curve) and 10 nm (red curve) (**b**). In all samples, the ALA solution concentration was 10^−2^ M.

Figure 10 highlights the dependence of the Raman spectrum intensity of ALA on the Au nanoparticles weight used to prepare metallic films. According to Figure 10a, the Raman spectrum intensity of ALA is enhanced as Au nanoparticles’ weight with a size of 50 nm increases. This fact is a consequence of the electromagnetic mechanism of the SERS effect, when the generation of localized surface plasmons at the metal/dielectric interface occurs [14]. According to Figure 10b, for the same weight of Au nanoparticles, a higher Raman intensity of ALA is found when the size of metallic nanoparticles is equal to 50 nm compared to those with a size of 10 nm. In the case of Au nanoparticles with a size of 50 nm, the EF of the ALA Raman line intensity situated in the spectral range 500–520 cm^−^^1^ was equal to 5.18 × 10^7^, a value superior to that reported in the case of Au nanoparticles with a size of 10 or 5 nm (Figure 9c,e).

### 3.4. HRTEM and AFM Studies

**Figure 11:**
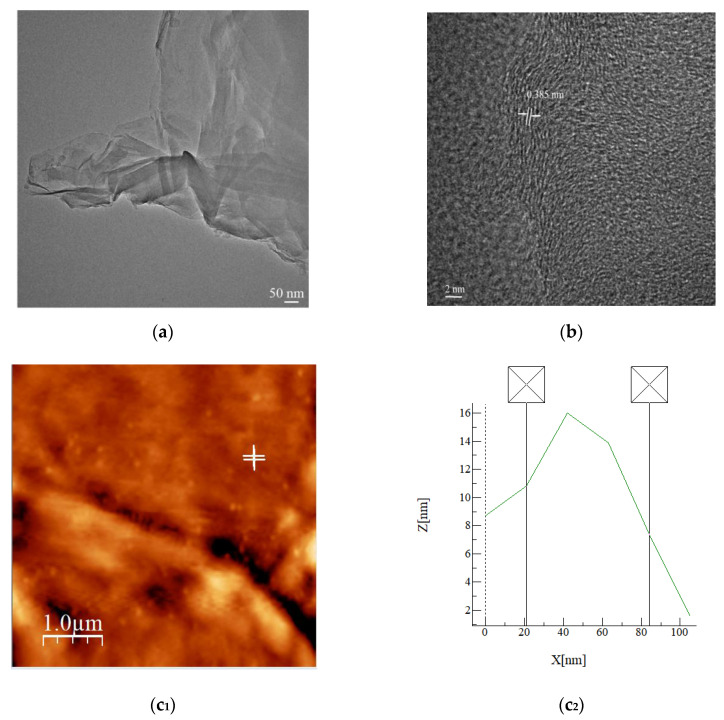

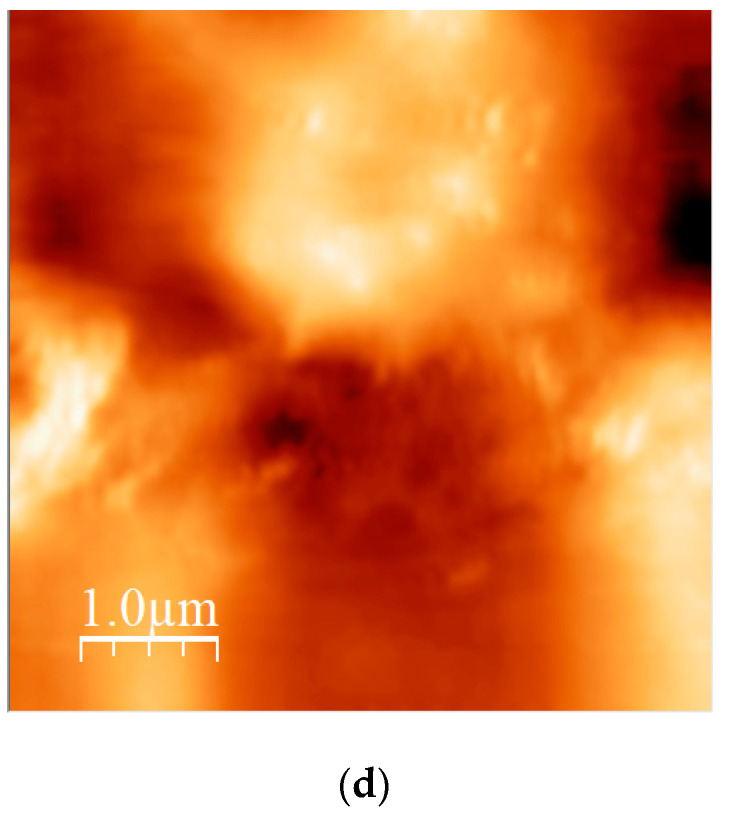
TEM (**a**) and HRTEM (**b**) images of the RGO sheets. The AFM image of the metallic film prepared from Au nanoparticles’ colloidal solutions with a size of 50 nm before (**c_1_**, **c_2_**) and after the deposition of the RGO sheets (**d**).

A short characterization of the RGO sheets by HRTEM and the film of Au obtained from the colloidal solutions of Au nanoparticles with a size of 50 nm before and after the adsorption of the RGO sheets by AFM is shown in Figure 11. Figure 11a shows the TEM image of the RGO sheets stacked with various folding. The HRTEM image of the RGO sheets (Figure 11b) highlights an interplanar distance d_002_ equal to 3.85 Å.

The AFM image of the films obtained by the evaporation of a 0.12 mL colloidal solution of Au, with a particle size of 50 nm, onto quartz substrates with an area of 1 cm^2^, are shown in Figure 11c_1_. As a consequence of the agglomerate and aggregate processes of the Au nanoparticles, the AFM images revealed that the gold nanoparticle diameter is ~60 nm (Figure 11c_2_). Two roughness parameters were calculated from the AFM scans for the Au film, i.e., the root mean square (RMS) and roughness average (Ra), with values equal to 18 nm and 13 nm, respectively. The deposition of the RGO sheets onto the Au film induced RMS and Ra values of 91 nm and 71 nm, respectively. Ra’s higher value in the case of the RGO sheets deposited onto the Au film caused their random orientation onto the metallic film surface (Figure 11d).

## 4. Conclusions

Using UV-VIS spectroscopy, new results are reported in this article concerning the photodegradation of ALA in the presence of Ag and Au nanoparticles. We have demonstrated that (i) the presence of excipients and additional active compounds to ALA, by exposure to UV light for 1 h, do not induce further changes once ALA has interacted with a colloidal solution of Ag and Au nanoparticles with sizes of 5 and 10 nm. (ii) The changes reported in the position and the relative intensities of Raman lines of ALA in the presence of Ag and Au nanoparticles with sizes between 5 and 10 nm, in contrast to the Raman spectrum of ALA powder, are explained by taking into account the chemical mechanism of surface-enhanced Raman scattering (SERS) spectroscopy. (iii) The photodegradation process of ALA that has interacted with metallic nanoparticles was inhibited in the presence of RGO sheets.
